# A Tool for the Assessment of Electromagnetic Compatibility in Active Implantable Devices: The Pacemaker Physical Twin

**DOI:** 10.3390/bioengineering12070689

**Published:** 2025-06-24

**Authors:** Cecilia Vivarelli, Eugenio Mattei, Federica Ricci, Sara D’Eramo, Giovanni Calcagnini

**Affiliations:** 1 National Centre for Artificial Intelligence, HTA and Techno-Assistance, Italian National Institute of Health, 00161 Rome, Italy; 2 Department of Cardiovascular, Endocrine-Metabolic Diseases and Aging, Italian National Institute of Health, 00161 Rome, Italy; eugenio.mattei@iss.it (E.M.); federica.ricci@guest.iss.it (F.R.); giovanni.calcagnini@iss.it (G.C.); 3 Department of Information Engineering, Electronics and Telecommunications, Sapienza University of Rome, 00185 Rome, Italy; deramo.1751057@studenti.uniroma1.it

**Keywords:** pacemaker, implantable defibrillator, electromagnetic interference, low-to mid-frequency magnetic field

## Abstract

Background: The increasing use of technologies operating between 10 and 200 kHz, such as RFID, wireless power transfer systems, and induction cooktops, raises concerns about electromagnetic interference (EMI) with cardiac implantable electronic devices (CIEDs). The mechanisms of interaction within this frequency range have been only partially addressed by both the scientific and regulatory communities. Methods: A physical twin of a pacemaker/implantable defibrillator (PM/ICD) was developed to experimentally assess voltages induced at the input stage by low-to-mid-frequency magnetic fields. The setup simulates the two sensing modalities programmable in PMs/ICDs and allows for the analysis of different implant configurations, lead geometries, and positions within a human body phantom. Results: Characterization of the physical twin demonstrated its capability to reliably measure induced voltages in the range of 5 mV to 1.5 V. Its application enabled the identification of factors beyond the implant’s induction area that contribute to the induced voltage, such as the electrode-tissue interface and body-induced currents. Conclusions: This physical twin represents a valuable tool for experimentally validating the mechanisms of EMI in CIEDs, providing insights beyond current standards. The data obtained can serve as a reference for the validation of numerical models and patient-specific digital twins. Moreover, it offers valuable information to guide future updates and revisions of international electromagnetic compatibility standards for CIEDs.

## 1. Introduction

In the last decade, we have witnessed a significant proliferation of new technologies operating with low- to mid-frequency magnetic fields (≤200 kHz). Radiofrequency identification (RFID) readers, wireless power transfer (WPT) systems, and induction cooktops are just some examples of technologies that generate magnetic fields and have become increasingly pervasive in our daily lives. These sources have been shown to cause transient malfunctions in pacemakers (PMs) and implantable cardioverter defibrillators (ICDs) [1,2,3,4,5]. Specifically, they produce electromagnetic fields (EMFs) with spectral content close to the operational bandwidth of these implantable devices, which may misinterpret ex-ternal signals as cardiac activity. The risks associated with such interactions include de-vice inhibition or the delivery of inappropriate therapy, which, in the worst-case scenario, could lead to serious injury or patient death [6,7,8].

Notably, many of these sources (such as EAS, RFID, and wireless charging systems) have only recently experienced widespread adoption, and for this reason, they are only partially addressed by current international regulations that define the minimum electromagnetic compatibility (EMC) requirements for PM and ICD manufacturers [6]. More-over, the feedthrough filters currently employed in PMs and ICDs to prevent electromagnetic interference (EMI) are specifically designed to operate at frequencies starting from the MHz range.

To date, numerical calculation tools are increasingly being established as essential support for assessing the risks faced by patients with PMs or ICDs exposed to EMFs. Not only in the scientific literature, but also in regulatory contexts, numerical analyses are becoming a valuable approach to demonstrating patient safety in the presence of EMFs. The development of “digital twins” is becoming more widespread across research and industrial applications, allowing for the modeling, simulation, monitoring, and prediction of their real-world counterparts [9,10,11]. However, every numerical study must first be experimentally validated, and in some cases, experimental measures are necessary to build the very foundation upon which numerical models are based.

For sources emitting magnetic fields up to 200 kHz, the interaction mechanism currently assumed to characterize interactions with PMs/ICDs is essentially based on Fara-day’s law of induction, applied to the loop formed by the implant area. While experimental evidence has shown that at frequencies below 10 kHz (particularly at 50–60 Hz, typical of power transmission systems) this assumption holds true [12,13,14], at higher frequencies it remains highly debated [12,15,16,17,18]. In this study, we propose the design, validation, and use of a “sensorized” physical twin of the PM/ICD implant—an electrical circuit capable of measuring the RMS signals induced by EMFs at the input stage of the implanted device, within the desired frequency range. This “physical twin” replicates the two sensing modalities of PM/ICD systems (i.e., unipolar and bipolar) and estimates the voltage induced as a function of the implant position within the body, as well as the characteristics and path of the leads. These data represent fundamental information to better understand the underlying mechanisms of interactions between EMFs and implants to carry on extensive EMC tests and to support the validation and verification of numerical models that will underpin the development of the patient-specific digital twin for individuals with PMs or ICDs.

## 2. Materials and Methods

The design of the physical twin was guided by a set of key requirements aimed at ensuring both functional robustness and experimental versatility:

- Compatibility with Commercial Leads: The device must interface with commercially available leads, both unipolar and bipolar, through the IS-1 standard, which is widely adopted by the majority of PM and ICD manufacturers.
- Operation within a Human Trunk Simulator: The system must be capable of operating while immersed in a saline solution that replicates the dielectric properties of biological tissues.
- Induced Voltage Logging: The device should continuously record the induced voltage at its input terminals in the range of 1mV-1V. The system should allow for voltage measurements between the lead tip and the metallic housing of the PM/ICD (unipolar mode) or between the two electrodes (tip and ring) of the lead (bipolar mode).
- Wireless Data Transmission: The system must support wireless transfer of recorded data to an external computer for real-time monitoring and storage.
- Frequency and Amplitude Range: The device should reliably measure sinusoidal signals across a frequency range of 10 kHz to 200 kHz, with amplitudes up to the reference levels specified by the ICNIRP guidelines (50–100 µT).
- Battery Operation: The system should be battery-operated to avoid any possible ground path which could affect the induced voltage.

### 2.1. The Physical Twin: Hardware Components

To meet these specifications, the physical twin of the PM/ICD was developed as an integrated circuit with Bluetooth connectivity, comprising the following components:

- RMS-to-DC Converter (LTC1968, Analog Devices, Wilmington, NC, USA): This component outputs a DC voltage proportional to the RMS value of the input signal. It supports both single-ended inputs (referenced to ground) and differential inputs, maintaining a stable gain across the 50 Hz to 500 kHz frequency range.
- Digitally Controlled Switches (ADG619, CMOS SPDT, Analog Devices, Wilmington, NC, USA): Two switches allow dynamic selection of the input to the RMS-to-DC converter. They enable switching between unipolar mode (single-ended measurement referenced to ground) and bipolar mode (differential measurement between two input signals).
- Integrated Microcontroller with Bluetooth Module (ISP1507-AX, Insight SIP, Sophia-Antipolis, France): This module manages the switch states, acquires data from the RMS converter via its ADC, and transmits the measured signals wirelessly to an external receiver.
- Power Supply (Two CR2032 Lithium Coin Cells): These batteries provide a nominal supply voltage of 3 V, ensuring full portability and compliance with the design requirements for battery-powered operation.

The circuit is housed within a sealed metal enclosure that replicates the chassis of an actual PM/ICD. Connection to the lead is established using a genuine silicone header from a single-chamber PM device, conforming to the IS-1 standard commonly adopted by PM/ICD manufacturers.

**Figure 1:**
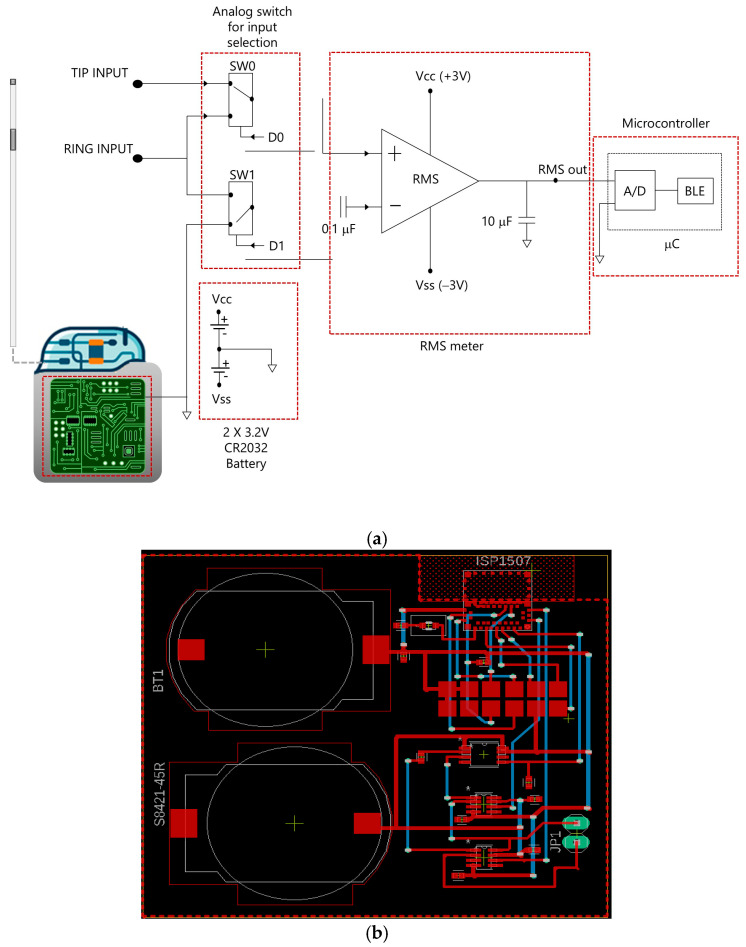
Block diagram (**a**) and board layout (**b**) of the PM/ICD physical twin.

To ensure realistic dimensions, all components were mounted on a compact PCB measuring 45 × 50 mm. The circuit schematic and PCB layout are shown in Figure 1. The board was designed as a double-layer PCB, with all components placed on the top layer. Special attention was paid to minimize the length of conductive traces and to maximize the separation between signal lines and power supply paths, thereby reducing susceptibility to external electromagnetic interference. Loop areas were minimized, and no components or copper planes were placed near the Bluetooth module to preserve optimal wireless communication performance.

**Figure 2:**
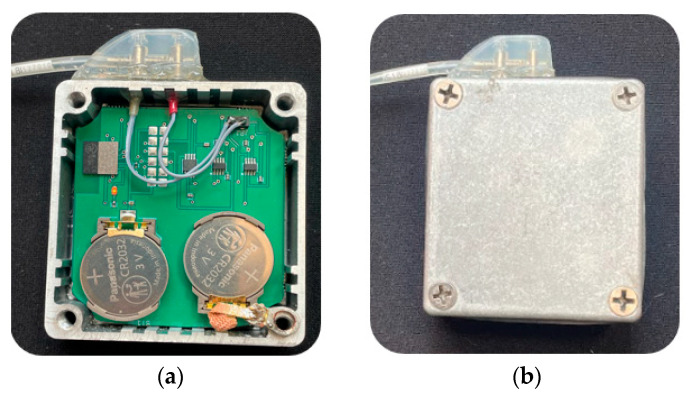
The PCB board housed in a metal enclosure (**a**). On the top of the metal enclosure there is the silicon header which allows lead connection with the circuitry. Top view of the physical twin with the metal enclosure is hermetically closed (**b**).

The PCB is housed in a waterproof metal enclosure with an O-ring seal (Figure 2), al-lowing safe immersion in torso simulators filled with saline solution. The metal case is electrically connected to the circuit ground and functions as the return electrode for unipolar measurements, effectively simulating the metallic housing of an actual PM/ICD. The silicone header includes two connectors for bipolar leads (tip and ring) and faithfully re-produces the IS-1 connection standard. The input impedance of the circuit, in the frequency range of interest, is approximately 10 kΩ, closely mimicking the electrical characteristics of a real PM/ICD [13].

### 2.2. The Physical Twin: Firmware

The firmware implemented on the microcontroller manages the two analog switches via digital control lines to select the input signal for the RMS meter. Three measurement modes are configured:

1. Unipolar tip measurement: The induced voltage is measured between the lead tip and the metal enclosure simulating the PM/ICD chassis.
2. Unipolar ring measurement: The induced voltage is measured between the lead ring electrode and the metal enclosure.
3. Bipolar measurement: The induced voltage is measured between the tip and ring electrodes of the lead.

In addition to managing the measurement modes, the firmware incorporates a custom Bluetooth protocol for wireless control and data transmission to an external PC. A dedicated graphical user interface (GUI) was developed in LabVIEW 2021 SP1 (National Instruments, Austin, TX, USA) to facilitate interaction with the system. This interface controls a commercial USB Bluetooth module (Bluegiga BLE(D)11x, Bluegiga, Duluth, GA, USA), issuing the specific commands required to operate the measurement board:

- Remote activation and pairing with the measurement board.
- Battery monitoring during continuous operation.
- Real-time data acquisition and visualization of induced voltage.
- On-the-fly switching between measurement modes.
- Monitoring of the measurement modality and power-down to conserve battery life.

To balance low power consumption and reliable communication (~1 m range), the Bluetooth profile was customized, ensuring efficient operation even when the device is immersed in saline solution simulators.

### 2.3. The Physical Twin: Performace Characterization and Validation

The following sections present the circuit performance characterization, focusing on resolution, linearity, and the influence of the lead on the measured voltage.

Subsequently, the physical twin was employed to measure the voltage induced by a homogeneous magnetic field, generated by a pair of Helmholtz coils operating at selected frequencies between 10 kHz and 200 kHz: 10 kHz, 20 kHz, 50 kHz, 100 kHz, 150 kHz, and 200 kHz. The setup consists of two rectangular Helmholtz coils (50 × 40 cm, six turns each), powered by a custom resonant circuit with an Arduino Nano controller (Arduino AG, Lugano, Switzerland). Series capacitors enable currents exceeding 2 A, producing magnetic fields above 100 μT at the target frequencies.

**Figure 3:**
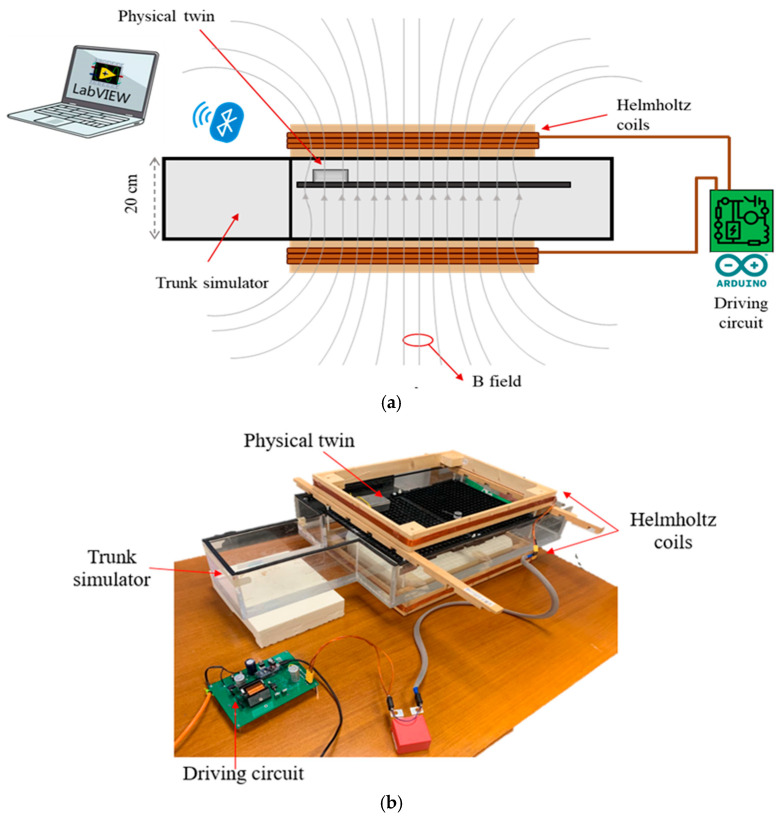
The exposure set-up used for the measurement with the physical twin: (**a**) schematic representation; (**b**) actual implementation.

The coils, spaced 20 cm apart, are positioned around a Plexiglass tank, filled with saline solution (conductivity: 0.2 S/m) to replicate human tissue dielectric properties over the 10–220 kHz range. Conductivity was calculated as a weighted average of torso tissue values [19]. Inside the tank, a supporting PVC grid holds the physical twin and the lead in place (Figure 3).

Induced voltages were measured on the physical twin, with the lead positioned at the center of the trunk simulator, as far as possible from its edges. Measurements were taken using two configurations:

- C-shaped configuration: The loop, formed by the lead, the metal case, and the connection through the saline solution between the lead tip and the case, resulted in a geometrical induction area of approximately 450 cm^2^.
- S-shaped configuration: The loop formed two opposite areas, yielding opposing contributions to the induced voltage, which resulted in a geometrical induction area of almost zero.

A commercial bipolar lead (GDT 4474/438-25S, 58 cm length, active fixation, Boston Scientific, Marlborough, MA, USA) was used. Measurements covered the selected frequencies in the range of 10–200 kHz and multiple field intensities, ensuring operation within the circuit’s linear range and avoiding saturation at high frequencies. All voltages were normalized by field intensity and frequency, allowing direct comparison across exposure scenarios.

## 3. Results

### 3.1. Physical Twin Chatacterization

The system’s resolution can be estimated from the characteristics of the analog-to-digital converter (ADC) used to acquire the RMS meter’s output signal. The ADC was set to sample the signal at a rate of 130 Hz, and it supports different gain settings. As a result, the system’s measurement range and resolution can be adjusted according to the expected amplitude of the measured signal. The ADC was configured for 12-bit conversion over a full scale of ±600 mV. For a given gain ***G***, the corresponding measurement range (*V_max_*) and resolution (*V_min_*) can be obtained as

(1) $$V_{max}=600\;\mathrm{mV}\cdot \frac{1}{G};\;V_{min}=\frac{\mathrm{mV}}{2^{12}}\cdot \frac{1}{G}$$

Table 1 summarizes the measurement settings that can be programmed in the physical twin.

In Table 1, the system’s accuracy is also reported. Accuracy measurements were obtained by short-circuiting the input of the physical twin (tip and ring connectors) and measuring the standard deviation (S.D.) using two averaging factors (8 samples and 16 samples).

The overall measurement range of the system must also consider the input characteristics of the RMS meter. To estimate the input–output response of the physical twin, a known signal at selected frequencies was injected into its input, using a setup consisting of a signal generator (SMB 100A, 9 kHz–3.2 GHz, Rohde and Schwarz, Munich, Germany) and a wideband amplifier (TOE 7608, Toellner Electronic Instrumente GmbH, Herdecke, Germany). The selected signal frequencies were 10 kHz, 20 kHz, 50 kHz, 100 kHz, 150 kHz, and 200 kHz. The signal amplitude was gradually increased from 0 V to approximately 2.5 V.

The input–output relationship is linear up to approximately 1.4 V, beyond which the output begins to saturate. Therefore, the system’s linear response range extends up to 1.4 V.

**Figure 4:**
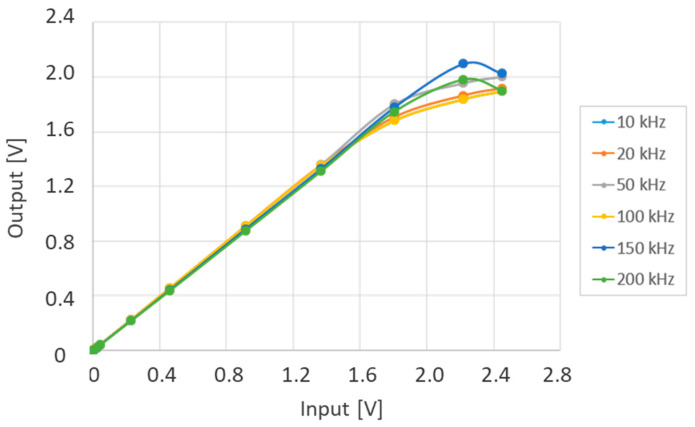
Input vs. output response of the physical twin.

The curves in Figure 4 were obtained by applying the signal between the tip input and the device’s metal enclosure. The same measurements were repeated with the signal applied between the ring input and the enclosure and between the tip and ring inputs. In all configurations, the recorded voltage displayed consistent behavior relative to the input signal amplitude.

**Figure 5:**
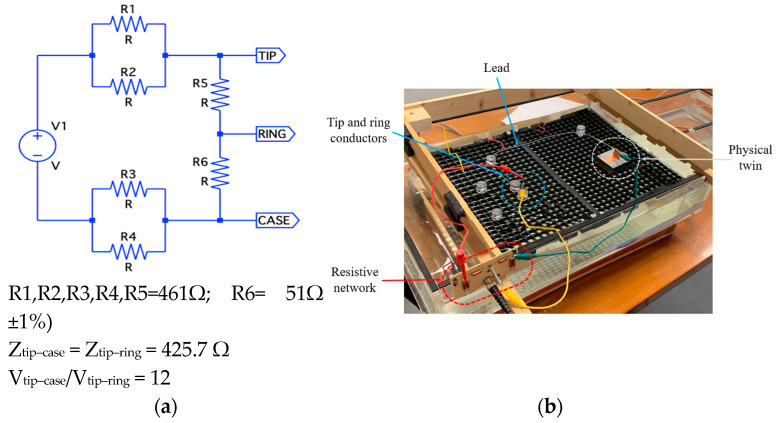
Resistor network (**a**) and test set-up (**b**) for physical twin characterization.

A second set of measurements was conducted with a lead connected to evaluate the lead’s potential contribution to the induced voltage within the frequency range of interest. The setup replicated the typical impedance conditions encountered by an implanted lead. Specifically, the lead electrodes (tip and ring) were connected to a resistor network (Figure 5a) designed to emulate the typical tip–ring impedance of a commercial lead (approximately 500–800 Ω). The network also ensured a 1:10 ratio between unipolar and bipolar voltages, as referenced in the literature [6,13].

The set-up included the torso simulator filled with saline solution (conductivity: 0.2 S/m) and a commercial PM lead (GDT 4474/438-25S, 58 cm length, active fixation, Boston Scientific, Marlborough, MA, USA). Both the lead and the device were immersed in the saline solution, with the exception of the electrode tip and the upper part of the device chassis (Figure 5b).

**Figure 6:**
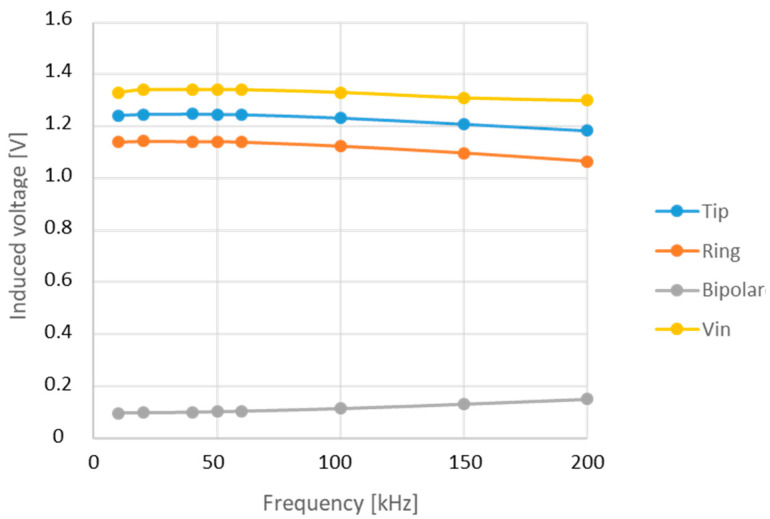
Voltage measured by the system when injecting a fixed signal (Vin) at the lead electrodes, at selected frequencies in the range of 10–200 kHz.

The voltages measured by the device at the selected frequencies, for a given input signal applied at V1 (Vin), are shown in Figure 6.

### 3.2. Measurement of the Induced Voltage

For each frequency selected within the 10–200 kHz range, the induced voltage measured by the physical twin was recorded at various magnetic field intensities. Table 2 summarizes the field strengths generated at each frequency. At frequencies above 50 kHz, given the geometry of the lead path used (450 cm^2^), field levels exceeding 50 µT resulted in induced voltages exceeding the linearity range of the system and could not be used.

**Figure 7:**
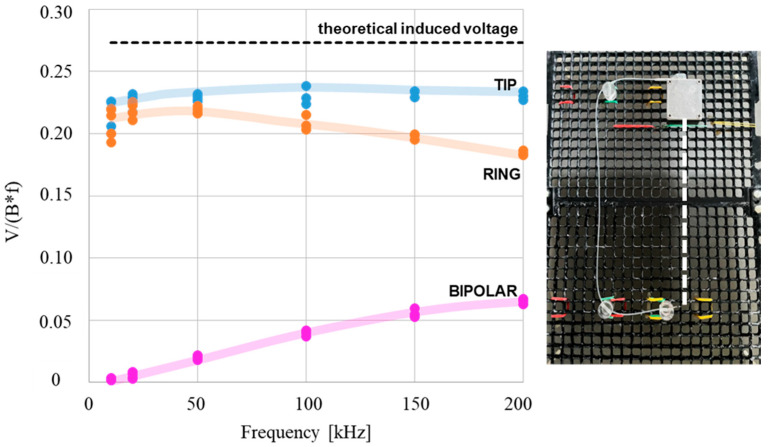
Normalized voltages measured by the physical twin for the C-shaped lead path across the three measurement modes (unipolar tip, unipolar ring, and bipolar). Each point on the graph corresponds to one of the tested field intensities. The black dashed line in the graph indicates the voltage value predicted by Faraday’s law of induction. The geometrical induction area is delimited by the C-shaped lead and the with dashed line connecting the lead tip and the metal case.

Figure 7 shows the induced voltages measured by the physical twin across the three measurement modes (unipolar tip, unipolar ring, and bipolar), with the lead arranged in a C-shaped configuration and positioned at the center of the trunk simulator, delimiting a geometrical induction area of 450 cm^2^. All voltages were normalized by field intensity and frequency. For a given frequency, each point on the graph corresponds to one of the tested field intensities. The dashed line indicates the voltage value predicted by Faraday’s law of induction, according to the relation *V* = 2*πfBA*, where *A* = 450 cm^2^ and *B* the magnetic field intensity.

**Figure 8:**
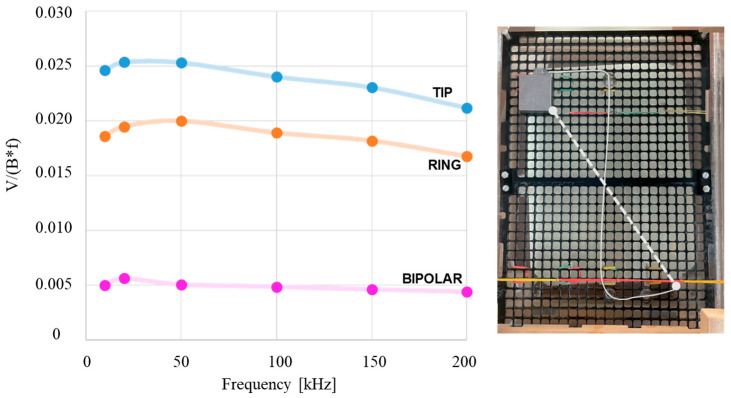
Normalized voltages measured by the physical twin for the S-shaped lead path, across the three measurement modes (unipolar tip, unipolar ring, and bipolar). The geometrical induction area is delimited by the S-shaped lead and the with dashed line connecting the lead tip and the metal case.

Figure 8 displays the induced voltages measured by the physical twin across three measurement modes (unipolar tip, unipolar ring, and bipolar) with the lead arranged in an S-shaped configuration. As depicted in the image illustrating the physical twin’s placement over the PVC grid, the lead forms two equivalent areas that contribute with opposite signs to Faraday’s law of induction. Consequently, the measured voltage is expected to be very close to zero. For this S-shaped lead configuration, all voltages were also normalized by field intensity and frequency.

## 4. Discussion

The conventional approach to assess the electromagnetic immunity of PM/ICD systems involves testing commercial devices under controlled exposure conditions, as specified by international standards, to evaluate deviations from expected operation when subjected to electromagnetic fields. These procedures typically aim to determine the threshold levels of electric or magnetic fields at which interference occurs. However, while effective for compliance verification, this methodology does not provide insights into the specific mechanisms of interaction between the external EMF and the device. As a result, it remains challenging to identify the system’s most vulnerable components—that is, the critical points responsible for EMI events. In addition, when EMI tests are performed on commercial devices, the need to cover samples from different manufacturers and techno-logical platforms available on the market makes this approach very time-consuming, and the results obtained are often less generalizable. Computational modeling, while powerful, requires detailed knowledge of both the electromagnetic field source and the internal architecture of the implantable device. This information is not always accessible and even when available may not allow for the accurate modeling of all physical processes involved in the voltage induction, such as interactions at the electrode–tissue interface.

The principal innovation introduced in this work is the design, validation, and ap-plication of a “sensorized” physical twin of the PM/ICD implant. This custom-developed electrical circuit can be connected to commercial PM/ICD leads. It replicates the functional input stage of the actual device and is capable of directly measuring the voltages induced by external electromagnetic fields within the relevant frequency range. The physical twin enables a detailed analysis of the induced signals at the device input under both unipolar and bipolar configurations.

Unlike conventional testing with commercial devices, the physical twin provides quantitative data on the effects of external electromagnetic fields and allows for the de-composition of contributions from individual system components. Specifically, it enables the evaluation of how factors such as lead path geometry, implant configuration, anatomical positioning within the body, and lead structural characteristics influence the system’s susceptibility to EMI.

Such a level of detail would not be achievable using a commercial PM/ICD device. The electrograms recorded and transmitted by commercial PM/ICDs, via proprietary communication protocols, are typically sampled at rates insufficient to capture the fast transients induced by external EM fields operating at frequencies of 10 kHz and above. Furthermore, these signals undergo substantial filtering, effectively removing critical information about high-frequency disturbances that may underlie device malfunctions. The physical twin developed in this study is an electrical circuit designed to measure the signals induced by EMFs on PM/ICD systems in the low-to-mid-frequency range of 10–200 kHz. Specifically, it records the induced voltages at the input stage of the device in both unipolar and bipolar sensing configurations. The physical twin provides a practical and immediate means to compare induced voltage measurements with the test levels specified by international regulations [6]. This capability is a key advantage of the platform proposed in this work, since, for frequencies below 385 MHz, immunity tests are specified in terms of conducted signal amplitude and modulation, which must be applied directly at the PM/ICD input stage. The frequency range for which the physical twin was designed to measure induced voltages aligns with that of many technologies increasingly present in both everyday and specialized environments. Examples of real-world sources operating in this range include RFID systems (125–134 kHz), wireless power transfer systems (80–100 kHz), induction cooktops (20–100 kHz), and muscular magnetic stimulation devices (1–20 kHz).

This tool is expected to significantly enhance current understanding of the interactions between EMFs and implanted devices by providing experimental evidence on how induced voltages are generated within the system. While at frequencies below 10 kHz the interactions between the magnetic field and the implant are well described by Faraday’s law of induction, recent studies suggest that as frequency increases, simple geometrical models (e.g., those based solely on the implant area) become insufficient to fully explain the observed phenomena. At higher frequencies, additional factors must be considered, including (i) currents induced in the body by external magnetic fields, independent of the presence of the implant itself; (ii) the specific interactions between the lead and the electromagnetic field at varying frequencies, including the effects at the electrode–electrolyte interface between the lead and surrounding tissues; and (iii) the internal characteristics of the PM/ICD circuitry.

One important implication of these findings concerns the assumed protective role of bipolar sensing configurations in PM/ICD implants. Current technical standards defining minimum EMF immunity requirements for PMs/ICDs [6] acknowledge that bipolar sensing offers enhanced protection against electromagnetic disturbances. This is primarily justified by geometric considerations on the Faraday’s induction law: the shorter distance between the sensing electrodes in the bipolar mode (approximately 2–4 cm between tip and ring) compared to the unipolar mode (approximately 20–25 cm between the lead tip and the device casing). Such considerations directly influence the amplitude of the test signals prescribed for EMC assessments of cardiac implantable electronic devices. According to the ISO 14117 standard [6], the test levels for unipolar configurations are derived from the European Commission Recommendation 1999/519/EC, which itself references the ICNIRP 1998 guidelines [20]. For bipolar configurations, however, international standards prescribe a reduction in test signal amplitude to 10% of that used for unipolar configurations. This reduction is based on the rationale outlined in EN 50527-2-1 [21], where the maximum voltage that could be induced in unipolar mode, estimated based on implant geometry and equivalent induction area, is considered to be at least an order of magnitude greater than that in bipolar mode. However, if factors beyond simple geometrical considerations—such as those that can be investigated by the physical twin proposed in this study—significantly contribute to the induced voltage, current testing protocols for bipolar sensing configurations may no longer be adequate to ensure patient safety under electromagnetic field exposures up to general public reference levels.

The characterization of the physical twin demonstrated its capability to adapt the measuring range and resolution to the specific characteristics of the signal expected to be measured. This feature allows the system to operate over a wide frequency range, compensating both for the linear increase in induced voltage that typically occurs as the frequency of the EMF increases, and for the gap—especially at lower frequencies—between unipolar and bipolar voltages. The system’s resolution, even at its maximum measurement range (less than 1 mV), is well suited to the typical sensitivity values that can be programmed in PM/ICD implants (typically 5 mV for unipolar sensing, 0.5 mV for bipolar sensing). This enables the system to potentially capture signal levels that may result in oversensing, undersensing, or inappropriate therapy delivery, particularly in devices operating with high sensitivity. However, to assess patient risk more systematically, the recorded voltages are primarily interpreted in light of the conducted signal amplitude thresholds defined by ISO 14117. As already stated, this standard provides frequency-specific voltage limits above which a risk of EMI cannot be excluded. When the induced voltage measured by the physical twin remains below these thresholds, the likelihood of adverse clinical effects is considered low. Conversely, if the recorded voltages exceed the standard thresholds, the data suggest a potential risk, warranting further investigation with actual devices or additional test scenarios.

The measurements performed by directly injecting a voltage signal into the lead demonstrated that the parasitic capacitances between the tip and ring conductors—co-axially arranged within the electrode lead—contribute to a low-pass filter behavior of the lead itself. However, the frequency-dependent response of the lead alone is insufficient to fully account for the pronounced differences in induced voltage observed across the 10–200 kHz range, as reported in previous studies [12,16,18].

Data obtained from the physical twin exposed to the magnetic field generated by the Helmholtz coils also highlight additional factors contributing to the induced voltage beyond the implant’s geometrical induction area alone. The measurements performed using the C-shaped lead configuration revealed that the unipolar voltage measured at the tip electrode is lower than what would be predicted by a straightforward application of Faraday’s law, and it shows limited variation with frequency. This discrepancy can be explained by the difference between the idealized and the actual return path geometry. In analytical and numerical models, the return path that closes the loop (connecting the lead tip and the device case) is assumed to be a straight segment, resulting in a well-defined, geometrical induction area. However, in the physical twin, this return path is a distributed trajectory within the phantom, constrained by the finite volume and the realistic anatomy. This leads to a smaller effective induction area, and consequently a reduced induced voltage. This effect has been noted in previous studies and is consistent with observations reported in related standards and literature [6,15].

Regarding the unipolar voltage measured at the ring electrode, we observed a progressively decreased value across the investigated frequency range, deviating increasingly from the theoretical predictions based on Faraday’s law of induction. Conversely, the bipolar-induced voltage increased with frequency, and the unipolar-to-bipolar voltage ratio declined sharply from approximately 80 at 10 kHz to nearly 1 at 200 kHz, in agreement with findings reported in [18]. This behavior is also consistent with the implant equivalent circuit model described in [16], which attributes the increase in bipolar-induced voltage to the combined effects of the implant’s internal circuitry and the electrode–tissue interface formed between the lead tip and ring electrodes. Other potential contributing factors include the effects of eddy currents and the influence of lead path design [12]. Further measurements and dedicated analyses are needed to fully characterize these phenomena.

Another factor that can influence the induced voltage at the PM/ICD input stage is the lead configuration and its position inside the trunk. While a comprehensive sensitivity analysis was beyond the scope of the present work, we tested two lead configurations simulating the two typical implant positions: one in the left pectoral region (C-shaped) and one in the right pectoral region (S-shaped). The latter results in a reduced geometrical loop area compared to the former and therefore lower susceptibility to electromagnetic induction. As expected, the induced voltages measured in the S-shaped configuration were significantly lower than those observed with the original C-shaped lead. Indeed, the S-shaped lead path generates two opposing induction areas, which contribute with opposite polarity to the induced voltage. Since these two areas were approximately equal in size, the resulting induced voltage was, for all tested frequencies and across all three measurement modalities, close to the resolution limit of our system. These results confirm the importance of lead path geometry in field coupling and demonstrate how the physical twin can serve as a flexible and practical platform for future investigations of patient-specific or clinically variable scenarios.

For future studies, the combined use of the physical twin proposed in this study and real PM/ICD devices could offer an efficient approach for assessing risks from EMF exposure. EMI assessments could then follow a stepwise approach:

1. Initial measurement of the voltage induced by the EMF source using the physical twin. This step assesses whether the measured value, at the frequency of interest, exceeds limits set by international standards.
2. If the induced voltage is below regulatory thresholds, the risk of EMI can be considered low.
3. If the induced voltage exceeds these thresholds, a potential risk of EMI events cannot be excluded. In such cases, additional measurements using actual devices should be carried out for further risk assessment.

## 5. Conclusions

The concept of developing a physical twin of the PM/ICD implant arose from the need to provide a practical and immediate means to compare induced voltage measurements with the test levels specified by international regulations [6], which define conducted signal amplitudes up to 385 MHz. In this way, the physical twin offers a rapid assessment of the risks associated with specific environments where EMF sources are present, eliminating the need to test samples from different manufacturers and technological platforms available on the market, a process that would otherwise be extremely time-consuming. It enabled a detailed characterization of how low-frequency magnetic fields—originating from technologies such as RFID systems, WPT systems, electronic surveillance (ES) gates, and induction cooktops—induce voltages at the input stage of the device. This tool provides valuable data not only for deepening the understanding of the interaction mechanisms between the implant and external electromagnetic fields, but also for informing potential future updates to international standards governing the EMC testing of pacemakers and ICDs.

By employing the physical twin, it is possible to quantitatively assess the influence of lead geometry, implant configuration, and positioning within the human body on device susceptibility. Specifically, this approach allows for a direct correlation between the intensity of external EMFs and the voltages induced at the input terminals of the implantable device. The developed tool has proven to be suitable to effectively investigate several phenomena, such as the effects of eddy currents and the contribution of the electrode–electrolyte interface, previously only hypothesized and never experimentally quantified.

Further refinements of the experimental setup will enable an even clearer under-standing of the role of these factors in the generation of interference, ultimately contributing to improved EMC assessment protocols and enhanced patient safety.

## Figures and Tables

**Table 1:** Measurement characteristics of the physical twin.

GAIN	Measurement Range (mV)	Resolution (mV)	Accuracy @ 8 Samp. Averaging (mV) ^1^	Accuracy @ 16 Samp. AVERAGING (mV) ^1^
1/4	2400	0.59	±1.6	±1.2
1/3	1800	0.44	±1.4	±1.0
1/2	1200	0.29	±0.8	±0.6
1	600	0.15	±0.6	±0.4
2	300	0.07	±0.4	±0.2
4	150	0.04	±0.4	±0.2

^1^ Accuracy is reported as ± 3 × S.D.

**Table 2:** Magnetic field values adopted to ensure operation within the measurement circuit’s linear range.

	16 μT	30 μT	50 μT	100 μT	150 μT	200 μT
10 kHz	×	×	×	×	×	×
20 kHz	×	×	×	×	×	×
50 kHz	×	×	×	×	×	
100 kHz	×	×	×			
150 kHz	×	×	×			
200 kHz	×	×	×			

## Data Availability

The original contributions presented in the study are included in the article, further inquiries can be directed to the corresponding author.

## Abbreviations

The following abbreviations are used in this manuscript:

PM	Pacemaker
ICD	Implantable Cardioverter/Defibrillator
CIED	Cardiac Implantable Electronic Device
RFID	Radiofrequency Identification
WPT	Wireless Power Transfer
EAS	Electronic Article Surveillance
EMF	Electromagnetic Field
EMC	Electromagnetic Compatibility
EMI	Electromagnetic Interference
